# Spontaneous Fluctuations in Sensory Processing Predict Within-Subject Reaction Time Variability

**DOI:** 10.3389/fnhum.2016.00200

**Published:** 2016-05-09

**Authors:** Maria J. Ribeiro, Joana S. Paiva, Miguel Castelo-Branco

**Affiliations:** ^1^Visual Neuroscience Laboratory, Faculty of Medicine, Institute for Biomedical Imaging and Life Sciences (IBILI), University of CoimbraCoimbra, Portugal; ^2^Institute for Systems and Computer Engineering, Technology and Science (INESC TEC)Porto, Portugal

**Keywords:** intra-individual variability, visual processing, reaction time, electroencephalography (EEG), event-related potentials (ERPs), choice-reaction time task, single trial EEG, independent component analysis

## Abstract

When engaged in a repetitive task our performance fluctuates from trial-to-trial. In particular, inter-trial reaction time variability has been the subject of considerable research. It has been claimed to be a strong biomarker of attention deficits, increases with frontal dysfunction, and predicts age-related cognitive decline. Thus, rather than being just a consequence of noise in the system, it appears to be under the control of a mechanism that breaks down under certain pathological conditions. Although the underlying mechanism is still an open question, consensual hypotheses are emerging regarding the neural correlates of reaction time inter-trial intra-individual variability. Sensory processing, in particular, has been shown to covary with reaction time, yet the spatio-temporal profile of the moment-to-moment variability in sensory processing is still poorly characterized. The goal of this study was to characterize the intra-individual variability in the time course of single-trial visual evoked potentials and its relationship with inter-trial reaction time variability. For this, we chose to take advantage of the high temporal resolution of the electroencephalogram (EEG) acquired while participants were engaged in a 2-choice reaction time task. We studied the link between single trial event-related potentials (ERPs) and reaction time using two different analyses: (1) time point by time point correlation analyses thereby identifying time windows of interest; and (2) correlation analyses between single trial measures of peak latency and amplitude and reaction time. To improve extraction of single trial ERP measures related with activation of the visual cortex, we used an independent component analysis (ICA) procedure. Our ERP analysis revealed a relationship between the N1 visual evoked potential and reaction time. The earliest time point presenting a significant correlation of its respective amplitude with reaction time occurred 175 ms after stimulus onset, just after the onset of the N1 peak. Interestingly, single trial N1 latency correlated significantly with reaction time, while N1 amplitude did not. In conclusion, our findings suggest that inter-trial variability in the timing of extrastriate visual processing contributes to reaction time variability.

## Introduction

Transient and rapid fluctuations in behavioral performance are a characteristic of human behavior, even when task characteristics are maintained constant (MacDonald et al., 2006). This intra-individual variability is thought to follow spontaneous moment-to-moment changes in brain state that affect stimulus processing as well as post-perceptual processes (Weissman et al., 2006). The reaction time to the onset of a visual stimulus is a well-studied behavioral measure that presents moment-to-moment fluctuations. Inter-trial reaction time variability is significantly related to variability in sensory cortex responses. In spite of its lower temporal resolution, this has mostly been studied in functional magnetic resonance imaging (fMRI) studies using tasks requiring visual selective attention (Weissman et al., 2006, 2009). Under these conditions, moments of slower reaction times are associated with brain activity patterns characterized by reduced pre-stimulus activity levels in frontal control regions including the anterior cingulate cortex and dorsolateral prefrontal cortex, reduced stimulus-evoked responses in the visual cortex, increased activity in the default-mode network (DMN), and increased post-stimulus activity in the anterior cingulate cortex (Weissman et al., 2006; Prado et al., 2011). In addition, slower trials are associated with reduced functional connectivity between the anterior cingulate cortex and dorsolateral prefrontal cortex, two regions involved in attentional control (Prado et al., 2011). These findings suggest that slower trials occur during transient periods where attention moved away from the target stimuli. This is reflected in the reduced amplitude of target-evoked blood-oxygen-level dependent (BOLD) contrast imaging responses in slower trials when compared with faster trials and increased amplitude of distracter-evoked responses (Weissman et al., 2009). Thus, the moment-to-moment fluctuations in brain state that underlie inter-trial reaction time variability affect the way the sensory cortices process incoming stimuli.

One possibility is that reduced efficiency in sensory processing will increase the time required for stimulus perception thus delaying the motor response. Another possibility is that reaction time variability arises mostly from fluctuations in the time required for decision, response preparation and execution (post-detection processing). Both possibilities can in fact coexist. The fact that a significant correlation between reaction time and activity in the visual cortex was observed in fMRI studies suggests that variation in stimulus perception underlies at least in part reaction time variability. However, the limited temporal resolution of fMRI data restricts our understanding of the temporal dynamics of these processes. On the contrary, electroencephalography (EEG) and magnetoencephalography (MEG) are two techniques that permit the non-invasive measurement of brain activity with temporal resolution in the order of milliseconds, thus allowing a clearer study of the temporal dynamics of evoked responses. Previous studies have shown that also scalp brain electric/magnetic signals show a relationship between single trial reaction time and visual evoked responses. Using MEG, Amano et al. (2006) suggested that extrastriate visual evoked responses explained partially the inter-trial reaction time variability (Amano et al., 2006). These correlations were found using simple reaction time tasks, where decision processes are minimized. Yet, theoretically the timing of stimulus processing in sensory cortices should also relate to reaction time in tasks requiring more complex decision processes like oddball tasks or choice-reaction time tasks. In fact, an earlier EEG study used a within-subject analysis to compare fast and slow trials of an oddball paradigm, and found a significant relationship between the amplitude and latency of the posterior average N1 visual evoked potential, originating from extrastriate visual areas, and reaction time (Bahramali et al., 1998). However, this study characterized the ERP amplitudes in a fixed time window and for pooled averages, and thus, it did not provide information on the temporal evolution of visual processing and its relation to reaction time at a single trial level. Nevertheless, this study suggests a relationship between N1 and reaction time variability. Source localization studies of visual evoked potentials suggest that the late N1 component, with latency around 200 ms, is generated within the ventral occipito-temporal cortex (Martínez et al., 2001; Di Russo et al., 2002), a cortical region implicated in shape/object identification. Accordingly, the visual N1 potential has been implicated in visual discrimination (Vogel and Luck, 2000; Philiastides and Sajda, 2006), and is modulated by spatial and feature-based attention (Hopf et al., 2004; Codispoti et al., 2006; Martinez et al., 2007). Given the link between moment-to-moment reaction time fluctuations and fluctuations in attention (Prado et al., 2011), reaction time variability is likely related also to fluctuations in the N1 potential. Yet, a recent similar EEG study using a 2-choice reaction time task did not find a significant difference between the averaged N1 amplitude of fast and slow trials (Ramchurn et al., 2014). Thus, the relationship between visual evoked potentials and reaction time inter-trial variability is still an open question. Furthermore, the timing of sensory processing might not be reflected on the amplitude of the VEP peaks. In fact, the work of Amano et al. (2006) suggests that the temporal integration of the peak was a better indication, than amplitude or latency, of how long it takes for the visual cortex to process the sensory input. Changes in the temporal dynamics of the VEPs, including time of onset, slope, duration, as well as amplitude and latency, might explain inter-trial reaction time variability. Thus, it is important to understand in further detail how the trial-by-trial fluctuations in brain state affect sensory cortical responses to better understand the origin of the occasional but unavoidable lapses in human performance reflected in the slowdown of the motor responses.

To study the relationship between trial-by-trial reaction time variability and neural processing, it is important to be able to extract neural activity data at the single trial level. Several methods have been proposed to improve extraction of single trial EEG activity signals. These involve extracting spatial components, thereby integrating EEG over electrodes rather than across trials (Parra et al., 2002; Makeig et al., 2004; Philiastides and Sajda, 2006; Saville et al., 2011; De Vos et al., 2012; Boutonnet and Lupyan, 2015). Philiastides and Sajda (2006) improved signal-to-noise ratio by using a linear discriminator that integrates EEG over electrodes, and identified the N1 as a discriminating component in single trial analyses able to distinguish images of faces from cars. Also studying the face discriminating ability of the EEG signal, De Vos et al. (2012) compared several methods for single-trial extraction [raw sensor amplitudes, regression-based estimation, bandpass filtering, and independent component analysis (ICA)], and concluded that ICA lead to a superior separation of the face discriminating component, the N1 peak. ICA separation of high density scalp EEG data is able to recover components that represent physiologically relevant processes originating from a single cortical source area thereby enhancing spatial definition and signal-to-noise ratio relative to scalp electrode data (Makeig et al., 2004; Onton et al., 2006). Therefore, we opted to use ICA as a method for modeling task-related single trial fluctuations in visual processing.

We investigated the relationship between reaction time and visual evoked responses, using EEG recordings acquired while participants were engaged in a 2-choice reaction time task, and two different analyses. First, as we were interested in determining the temporal profile of this relationship, we studied the link between single trial ERP amplitudes and reaction time independently at each time point, in a within subject analysis. Second, we measured the single trial N1 peak latency and amplitude and studied the relationship between these single trial measures and reaction time.

## Materials and Methods

### Participants

Eighteen participants were included in this study (age range, 21–32 years; mean age 25 years; 3 left-handed; 8 female). All participants had normal or corrected to normal vision, and no history of learning, developmental, cognitive, neurological or psychiatric problems.

The study was conducted in accordance with the tenets of the Declaration of Helsinki and was approved by the Ethics Committee of the Faculty of Medicine of the University of Coimbra. Written informed consent was obtained from the participants, after explanation of the nature and possible consequences of the study.

### Visuomotor 2-Choice Reaction Time Task

**Figure 1:**
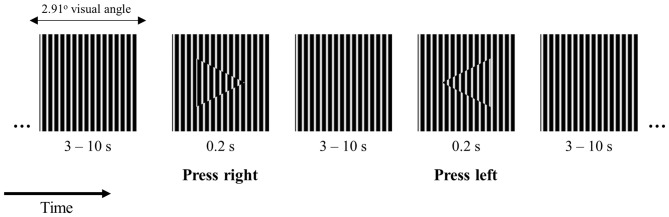
**Schematic illustration of the 2-choice reaction time task design and visual stimuli**.

We designed a 2-choice reaction time task where participants were instructed to fixate the center of a small square (side length −2.91 degrees of visual angle) filled with a vertical square grating pattern (Figure 1). The square was presented on the center of the screen on a gray background with luminance of 4 cd/m^2^. At intervals between 3 and 10 s randomly distributed, an arrowhead pointing to the left or to the right appeared on the center of the square for the duration of 0.2 s. The arrowheads were filled with the same vertical square grating pattern as the background shifted in phase as shown in Figure 1. The task was chosen in order to minimize working memory requirements and speed-accuracy tradeoffs while maintaining the condition for overt attention on the visual stimulus (this stimulus configuration made the target easily detectable but required overt attention). The stimuli were presented in the center of a Dell LCD computer monitor, with 56 cm (22”) diagonal viewable image size, set to a resolution of 1680 × 1050 pixels, and a refresh rate of 60 Hz. The participant’s head was positioned 60 cm from the computer screen on a chin rest to minimize head movements. Participants were instructed to press a button on the keyboard as quickly and as accurately as possible indicating the arrowhead direction with their left or right index fingers, respectively.

To allow subjects to rest, the task was divided into nine blocks of 4 min each, separated by periods of 15 s of fixation during which a white fixation dot was presented on the same gray background. In addition, the experiment was divided into three runs of three blocks each, allowing the participants to have three self-paced breaks.

### EEG Acquisition and Analysis

EEG signal was recorded using a 64-channel Neuroscan system with scalp electrodes placed according to the International 10–20 electrode placement standard and with reference between the electrodes CPz and Cz and ground between FPz and Fz. Acquisition rate was 1000 Hz. Vertical and horizontal electrooculograms were recorded to monitor eye movements and blinks. A trigger pulse was generated at the onset of each stimulus and at every button-press. Electrode positions were measured using a 3D-digitizer Fastrak (Polhemus, VT, USA) for accurate source analyses.

EEG data analysis was performed with Analyzer 2.0 from Brain Products GmbH and Matlab (The Mathworks Company Ltd) using the EEGLAB toolbox version 13.4.4 (Delorme and Makeig, 2004). The EEG data was first pre-processed in Analyzer: bad channels were excluded and interpolated; data re-referenced to average reference; downsampled to 256 Hz; and bandpass filtered using the Butterworth Zero Phase Filter with cutoff frequencies of 1 and 100 Hz and attenuation of 12 dB/octave. The data were then exported to Matlab and further analyzed with the EEGLAB toolbox. The continuous data were visually inspected and segments containing considerable muscle artifacts were removed before being submitted to extended Infomax ICA (Bell and Sejnowski, 1995) to find components of interest associated to the presentation of the visual target stimuli. Interpolated channels were excluded from ICA analysis. We used the default parameters of the binica program in EEGLAB. The independent components (ICs) activity data were cut into segments locked with the stimuli onset from −200 ms until 1000 ms after stimulus onset, and separated into left correct trials and right correct trials. Average baseline activity (set from −200 ms to stimulus onset) was removed from each trial. Next, we inspected the spatial, spectral, and temporal properties of each IC to identify those components corresponding to non-brain sources: eye blinks, lateral eye movement, muscular artifacts, single-channel artifacts, and high frequency line noise. These were excluded from further analyses. ICs related to early visual processing were manually selected based on the inspection of the scalp topography and ERP. First, we selected the ICs that showed posterior (occipitotemporal) topography and an N1 peak with latency around 200 ms in their average ERP. For some subjects, more than one component had such characteristics. In these cases, we chose for analysis the component that contributed the most to the average early ERP (between 50 and 250 ms after target onset) of the dataset, except for subject S16 where the component presenting the clearer N1 peak at 200 ms (more consistent with the components from the other subjects) explained slight less variance than another component with an earlier broader peak. The selected components were also in general the ICs presenting the N1 peak with higher absolute amplitude. This selection process is illustrated in the Supplementary Figure 1 that presents all the components that had posterior topography and an N1 peak in their IC ERP, with the selected component highlighted. In subject 6, back projecting the component of interest to a posterior channel revealed that its activity was reversed in polarity. Thus, for all the analysis, we inverted the component polarity to be compatible with the channels ERP. On average, the projection of our selected visual ICs into channel space accounted for 53% (SD 24%), for left trials, and 53% (SD 23%), for right trials, of the amount of the variance of the whole-channel EEG time series between 50 and 250 ms after stimulus onset.

### Single Trial IC ERPs’ Amplitude/Reaction Time Correlations

#### Time Point by Time Point Correlation Analyses

These analyses were carried out using Matlab (The Mathworks Company Ltd). For each subject, we computed the Pearson correlation coefficients between single-trial reaction time and single-trial component ERP values for each time point between 50 and 600 ms after target onset, corresponding to 142 data points. Our aim was to study early visual processing, thus, the analysis window chosen included the visual evoked response of interest, starting after 50 ms. We were only interested in the neural processing that occur before the button press and thus could affect its timing. Therefore, we only analyzed the ERP up to 600 ms, as at that time point the button press had already occurred in the majority of trials. For this analysis, extremely slow trials with reaction time values above 1 s were dismissed—these occurred very rarely in only four trials in three subjects. Group level significance of within participant correlations was tested statistically by using a one-sample *t*-test to determine whether the group mean of the correlation coefficients for each time point was significantly different from 0. Given that the sampling distribution of Pearson’s *r* is not normally distributed, the *t*-test analysis was preceded by Fisher *r*-to-*z* transformation. Correction for multiple comparisons was achieved using the “tmax” method for adjusting the *p* values of each variable for multiple comparisons (Blair and Karniski, 1993; Groppe et al., 2011). Like Bonferroni correction, this method adjusts *p* values in a way that controls the family-wise error rate. However, the permutation method is more powerful than Bonferroni correction when different variables in the test are correlated as is the case of temporally adjacent EEG data points (Groppe et al., 2011). Note that the number of comparisons was decreased by the downsampling of the EEG data to 256 Hz. Although our downsampling strategy was not related with an explicit goal of decreasing the number of multiple comparisons, it reduced serial correlations while maintaining the temporal resolution of the findings as most ERP events will happen for at least 10 ms.

#### Single Trial Latency and Amplitude Correlation Analyses

For single trial latency and amplitude measurements, the N1 peak was identified automatically using Matlab, as the minimum value between 100 and 350 ms after target onset. Amplitude and latency were measured at that time point. Trials with measured peak latency at the beginning or the end of the peak search window were excluded from analysis as these were not likely to be associated with a local minimum, as has been done before in single trial analyses (Saville et al., 2015b). Besides measuring the N1 amplitude in relation to the pre-stimulus baseline, we also measured N1 peak-to-peak amplitude in relation to the maximum positive amplitude value between 50 ms after stimulus onset and the N1 latency (P1). Low signal-to-noise in single trials might particularly affect baseline amplitude levels. Peak-to-peak measurements should be less sensitive to noisy baselines than absolute amplitude and thus might provide a more adequate measure of single trial peak amplitude.

For correlation analyses between reaction time and single trial peak amplitude and latency, we used a robust correlation technique, the Pearson’s skipped correlation (Wilcox, 2012), as implemented in the robust correlation Matlab toolbox (Rousseeuw, 1984; Pernet et al., 2013). Skipped correlations minimize the effects of bivariate outliers by taking into account the overall structure of the data. Notably, Pearson’s skipped correlation is a direct reflection of Pearson’s *r*. In single trial analyses, accounting for outliers is particularly important, given that peak detection is likely to fail in a certain percentage of trials.

For each subject, we computed the Pearson’s skipped correlation coefficients between reaction time, and single trial absolute peak amplitude, peak-to-peak amplitude, and latency. Group level significance of within participant correlation coefficients was tested by determining whether the group mean of the correlation coefficients was significantly different from zero with one-sample *t*-tests, after Fisher *r*-to-*z* transformation. We used Bonferroni correction to correct for multiple comparisons, thereby decreasing the critical *p*-value for significance to 0.008, corresponding to six comparisons.

#### Simulation of the Impact of N1 Latency Jitter on Average N1 Amplitude

In order to investigate the impact of N1 peak single trial latency variance on the N1 peak amplitude of the average ERPs, we run a simulation where we mimicked the single trial latency variability while keeping the single trial N1 amplitude constant. Single trials were simulated in the following manner. For each subject, we used the waveform of the subject specific average component ERP. For each trial, the individual waveform was shifted horizontally so that the N1 peak latency equaled the latency measured at each single trial for that subject. The trials with the shifted waveforms were then sorted according to reaction time, divided in tertiles, and averaged to simulate the ERPs of fast, median and slow tertiles. We measured the N1 peak amplitude as the minimum value between 100 and 350 ms of the fast and slow tertile ERPs and calculated the percentage decrease in amplitude observed in the slow tertile ERP in relation to the N1 amplitude observed in the fast tertile ERP. The equivalent percentage decrease in amplitude was also calculated in the empirically obtained individual tertile-split ERPs. Statistical comparison between simulated and empirically obtained percentage of amplitude decreases was performed using repeated measures analysis of variance (ANOVA) with trial type (left vs. right) and method (simulated vs. empirical results) as within-subjects factors. This analysis was performed with IBM SPSS Statistics version 22 Software.

### Independent Component’s Source Localization by Equivalent Dipole Modeling

In order to obtain the approximate source localization for the cluster composed of the selected ICs, we used the DIPFIT toolbox in EEGLAB (http://sccn.ucsd.edu/wiki/A08:_DIPFIT). We determined for every subject the location of the equivalent dipoles whose scalp projections most resembled the observed IC scalp distribution. ICs were fitted with equivalent current dipole models using each individual’s recorded electrode locations fitted to a template boundary element head model, and then localized in the template brain. ICs with bilaterally distributed scalp maps (subjects 3, 4, 6, 7, 8, 10, 11, and 14) were fitted with a dual equivalent dipole model with a positional symmetry constraint. The other subjects’ ICs were fitted with single dipole models. The residual variances (mismatch between the component scalp map and the model dipole projection) associated with the dipoles obtained were 6% on average (between 2 and 12%).

In order to compute the left hemisphere group-mean equivalent dipole location, we averaged the left hemisphere equivalent dipoles coordinates from unilateral and bilateral models. Accordingly, single dipoles belonging to unilateral and bilateral models localized on right hemisphere were averaged and the right hemisphere group-mean dipole location was determined.

## Results

### Behavioral Results

Participants responded correctly in the majority of trials with only small percentages of misses (1.0%) and incorrect responses (2.7%). As expected, reaction time fluctuated from trial to trial with average fastest responses just over 300 ms, and slower responses around 800 ms (Table 1).

**Table 1:** **Behavioral results**.

		Reaction time (ms)		
	Average number of trials per subject	Median	Standard deviation	Range (min–max)
Correct left responses	108	480	80	344–839
Correct right responses	108	467	77	323–775

*Number of correct trials, and reaction time measures for correctly responded left and right trials, averaged across subjects*.

### Electrophysiological Results

#### Relationship between Independent Components’ ERPs and Reaction Time

**Figure 2:**
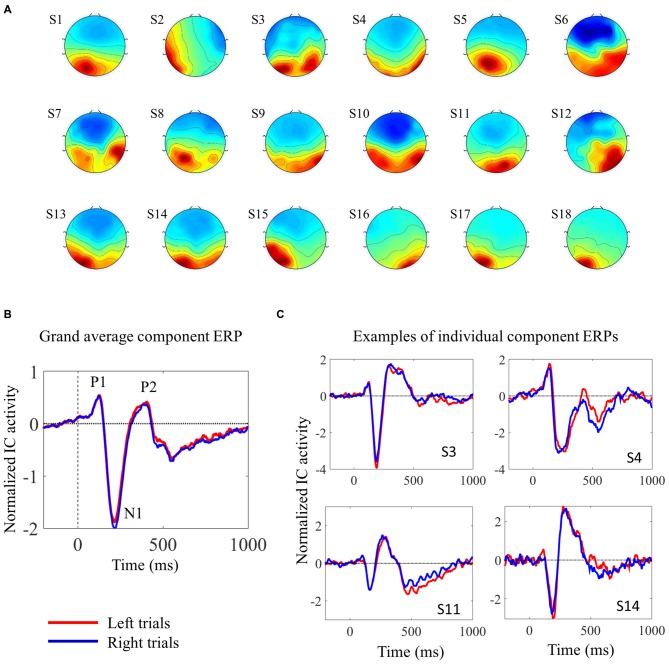
**Description of the selected independent components (ICs). (A)** IC scalp topographies of the selected visual ICs in the 18 individual subjects. **(B)** Shows the grand average IC event-related potential (ERP), locked with target onset, from 18 subjects. **(C)** Shows four examples of individual component ERPs, locked with target onset, from four participants **(C)**. In **(B,C)** red and blue lines represent activity elicited by left and right pointing targets, respectively. The visual ERPs elicited by the two different targets overlap almost completely.

We used ICA to analyze the EEG data from the 2-choice reaction time task. EEG signals from scalp electrodes comprise the sum of EEG activities originating in several different cortical areas. ICA is able to separate the activity from distinct sources, thereby facilitating the study of trial-by-trial fluctuations in the neural response of a single cortical area (Makeig et al., 1999; De Vos et al., 2012). We were interested in visual processing, in particular. In each subject, we were able to identify one component that contributed considerably to the early visual evoked response. For the subsequent analyses, only trials associated with correct responses were used, and trials were analyzed separately according to the target (left pointing arrowhead or right pointing arrowhead). The topographies and the ERPs from the individual components are shown in Figure 2. Figure 2C shows four individual IC ERPs examples. Although the presence of the early peak P1 was variable across subjects, the negative N1 peak could be consistently identified in all subjects.

**Figure 3:**
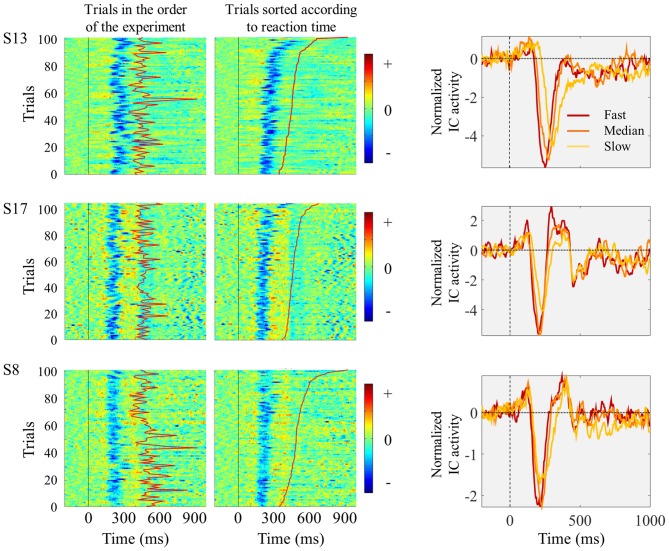
**Three individual examples showing trial-by-trial changes in ICs ERPs locked to the presentation of the left pointing target.** Each horizontal line of graphs represents data from one single subject. Left and middle columns show single-trial individual ICs ERP images time locked to target onset (marked by vertical black straight line at time 0). The moment of button press is represented by the red line between 300 and 900 ms after stimulus onset. In the left column, trials are presented in the order they appeared in the experiment. In the middle column, trials were sorted according to reaction time, with fastest responses appearing in the bottom. No vertical smoothing was applied. On the right column, the graphs represent IC ERPs divided in three quantiles according to reaction time. Red lines represent the ERPs obtained by averaging the one-third of trials with fastest responses; orange lines correspond to the one-third of trials around the median reaction time; and the yellow lines correspond to the one-third of trials with slowest responses.

The relationship between single trial visual ERPs and reaction time is exemplified in Figure 3 displaying the ERPs elicited by the left pointing target, from three sample participants. In comparison to faster trials, slower trials were associated with average ERPs with smaller amplitude, increased latency and longer duration.

**Figure 4:**
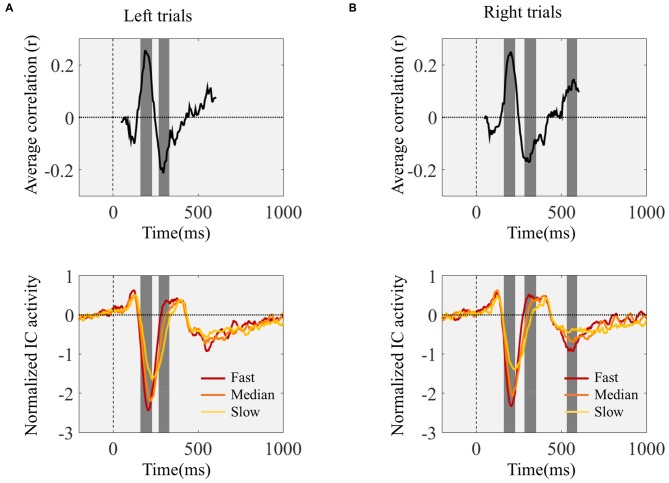
**Time point-by-time point correlation analysis between IC ERPs activity values and reaction time.** Top graphs show the Pearson’s correlation coefficients (*r*) for each IC ERP time point between 50 and 600 ms after target onset. The gray background highlights the time windows where the correlation coefficients were significantly different from zero. The bottom graphs present the grand-average IC ERPs. Before averaging across subjects the trials were divided into three quantiles according to reaction time. Red lines correspond to the one-third fastest trials; orange lines represent the one-third of trials corresponding to the reaction times around the individual medians; and the yellow lines represent the one-third slowest trials. **(A,B)** show the correlation coefficients and IC ERPs time locked with the onset of the left **(A)** and right **(B)** pointing targets.

We studied the temporal profile of the within-subject relationship between ERP amplitude and reaction time by determining the correlation coefficients between reaction time and ERP amplitude for each time point, between 50 and 600 ms after stimulus onset. We performed this analysis independently for each participant and determined group level significance by determining the time points for which the coefficients were significantly different from zero. This analysis revealed several time windows showing a significant relationship between ERP amplitude and reaction time: left trials 175–222 ms and 281–320 ms; right trials 175–222 ms, 296–339, and 546–577 ms (Figure 4). The first two time windows corresponded to the N1 peak.

**Figure 5:**
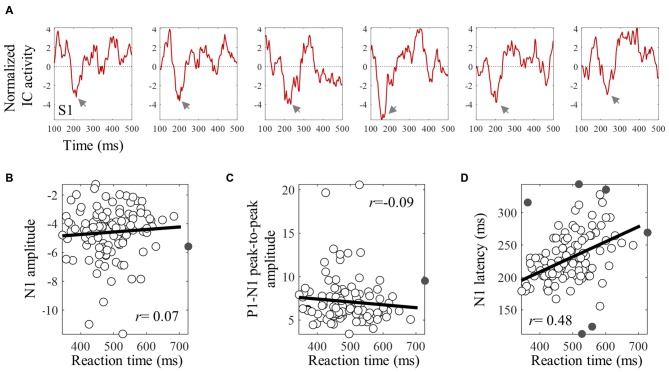
**An individual example of the within-subject relationships between reaction time and single trial N1 peak amplitude and latency. (A)** Shows six examples of single trial ERPs time locked with the onset of the target, for subject S1. Gray arrows point to the single trial N1 peak. **(B–D)** Show skipped correlations between reaction time and N1 amplitude **(B)**, P1-N1 peak-to peak amplitude **(C)**, and peak latency **(D)**. Gray points are bivariate outliers detected using the boxplot rule (Pernet et al., 2013). These were excluded from the correlation analysis.

**Figure 6:**
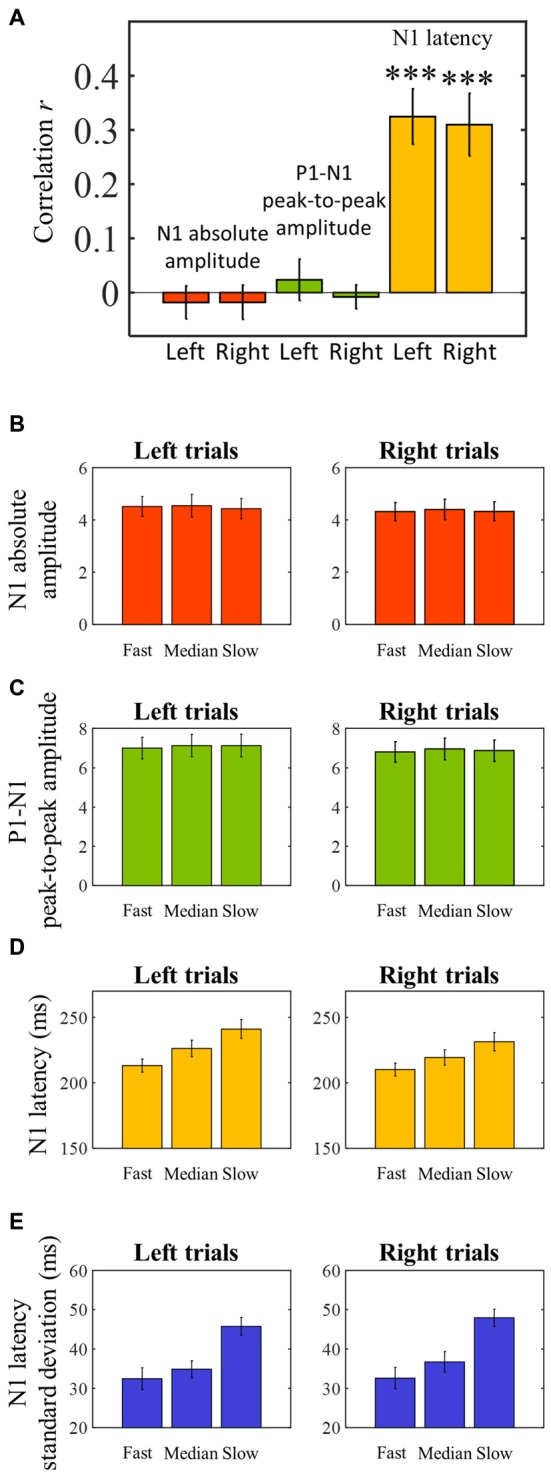
**Pearson’s skipped correlation coefficients between single trial N1 peak characteristics and reaction time—group level analysis. (A)** Across subjects’ mean skipped correlation *r* regarding the correlation analyses between reaction time and absolute N1 amplitude for left and right trials, P1-N1 peak-to-peak amplitude, and N1 latency for left and right trials. ***P < 0.0001. **(B–D)** For illustration purposes, single trial latency and amplitude measurements were averaged to create “jitter-free” amplitude and latency measurements for each subject (median single-trial amplitude and latency) for each reaction time tertile. The graphs represent across subjects’ mean N1 absolute amplitude **(B)**, P1-N1 peak-to-peak amplitude **(C)**, and latency **(D)** values for each reaction time tertile. **(E)** Single trial within-subject N1 latency standard deviation for each reaction time tertile averaged across subjects. Error bars represent across subjects’ ± 1 standard errors.

The averaged ERPs shown in Figures 3, 4 suggest that both peak amplitude and latency co-vary with reaction time. We tested this hypothesis by investigating directly the link between reaction time and single trial peak amplitude and latency. This analysis was made possible because in our component activity data the N1 peak was highly reliable and visual inspection suggested that it could be detected in the majority of trials. Examples of single trials from one participant are presented in Figure 5A, showing trial-by-trial variability in peak amplitude and latency. An individual example of the within-subject relationship between peak latency/amplitude and reaction time is displayed in Figures 5B–D. These graphs present the within-subject linear regressions between reaction time and N1 peak amplitude (Figure 5B), P1-N1 peak-to-peak amplitude (an alternative measure of N1 amplitude that might be more robust at the low signal-to-noise level of single trials; Figure 5C), and N1 latency (Figure 5D). These results suggest a positive relationship between reaction time and peak latency, and no clear relationship between reaction time and peak amplitude. These associations were confirmed at the group level. Group level correlation averages are presented in Figure 6A, and correlation results and group statistics are presented in Table 2. The correlation coefficients between reaction time and N1 latency were found to be significantly higher than zero (longer reaction times were associated with longer latencies), while the correlation coefficients between reaction time and N1 absolute amplitude or P1-N1 peak-to-peak amplitude were not significantly different from zero (Table 2). Thus, trial-by-trial differences in peak latency predicted reaction time, while peak amplitude did not. Figures 6B–D illustrate these relationships by displaying the average of the single trial amplitudes and latencies in each reaction time tertile representing the one third fastest, median and slowest trials. While single trial amplitude values did not change across tertiles (Figures 6B,C), latency values increased with increasing reaction time (Figure 6D).

**Table 2:** **Correlation results between single trial N1 peak amplitude/latency and reaction time**.

	N1 absolute amplitude		P1-N1 peak-to-peak amplitude		N1 latency	
	Left	Right	Left	Right	Left	Right
Average ***r***	−0.02	−0.02	0.02	−0.01	0.32	0.31
(min/max)	(−0.21/0.23)	(−0.29/0.28)	(−0.28/0.41)	(−0.28/0.13)	(0.07/0.71)	(0.08/0.79)
*P* value	0.56	0.58	0.54	0.72	4.8e^-6^	3.6e^-5^

*Averager (min/max) – across subjects average of correlation r values and across subjects minimum and maximum r values; p value—relative to the one-sample t-test testing if r values were significantly different from 0*.

#### Impact of Latency Jitter on N1 Peak Amplitude

**Figure 7:**
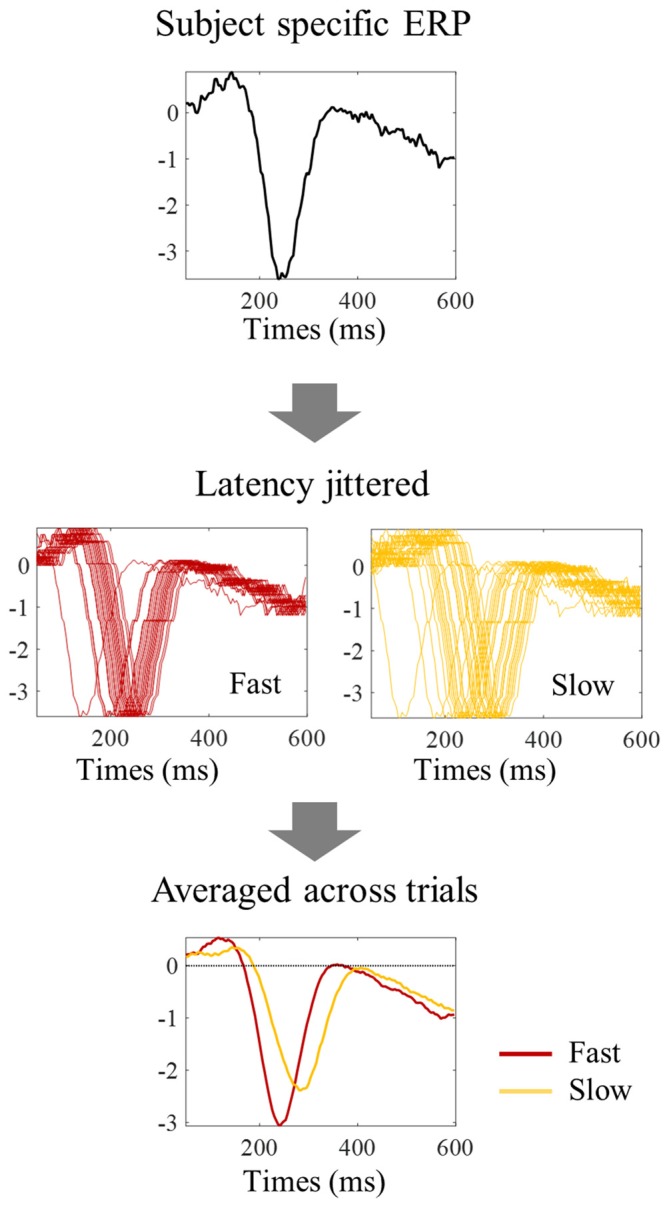
**Procedure to evaluate the impact of latency jitter on the N1 amplitude of averaged ERPs.** Each subject specific ERP waveform was shifted horizontally to match the N1 latency value of each individual trial within the fast, median, and slow group of trials, thereby keeping single trial N1 amplitude constant. The trials within each reaction time tertile were then averaged to simulate the tertile split ERPs. The higher latency jitter within the slow tertile resulted in an N1 peak with smaller amplitude in the averaged ERP.

**Figure 8:**
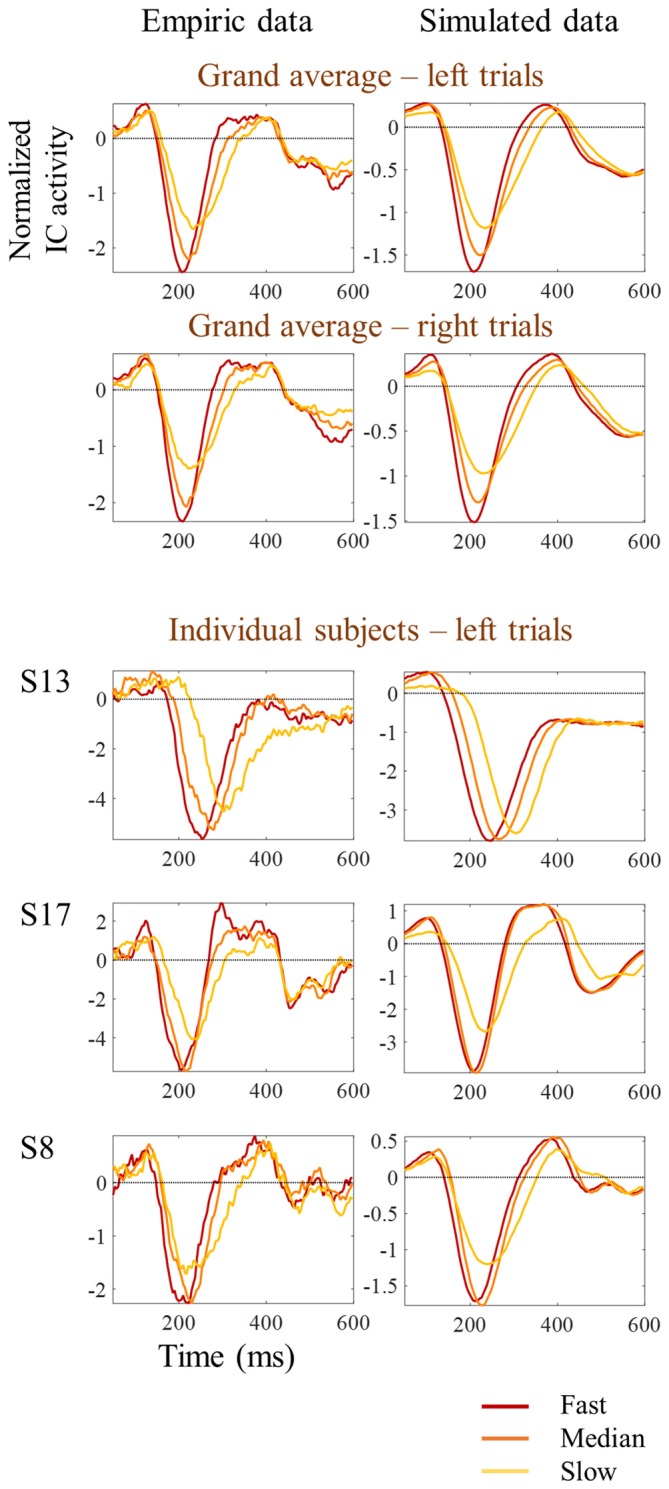
**Comparison between empirical and simulated latency jitter results.** Empiric reaction time tertile split ERPs are presented on the left, while simulated ERPs are presented on the right. Red lines represent the ERPs obtained by averaging the one-third of trials with fastest responses; orange lines correspond to the one-third of trials around the median reaction time; and the yellow lines correspond to the one-third of trials with slowest responses. Grand average ERPs are on top. Three individual examples corresponding to the subjects shown in Figure 3 are presented at the bottom.

One possibility is that the differences in amplitude observed in the tertile-split ERP averages illustrated in Figures 3, 4 were a consequence of the higher N1 latency variance within slowest tertile, in line with the characteristic higher variance of reaction time in the slower third of the trials. Accordingly, the within-subject standard deviation of the N1 single trial latency increased across tertiles (Figure 6E). In order to check if the higher latency variance could explain the lower amplitude observed in the slower tertile, we run a simulation where we reproduced the single trial latency variance while keeping the single trial N1 peak amplitude constant. Figure 7 illustrated the simulation process in an individual example. Figure 8 shows examples of the average tertile-split ERPs of the simulated data on the right and the empirically obtained waveforms on the left. The empirical and simulated grand average waveforms were strikingly similar. In the simulated data, the amplitude of the grand average N1 peak of the slow trials was 33% smaller than the amplitude of the fast N1 peak (averaged across left and right trials). In comparison, in the empirical data, the grand average N1 peak of the slow tertile was 36% smaller than the N1 peak amplitude of the fast tertile. We calculated the percentage decrease in N1 amplitude in the slow tertile compared with the fast tertile for each subject, and compared the simulated and the empirical results. The average across subjects (± standard deviation) decrease in N1 amplitude in simulated data was 18 ± 13% for left trials and 18 ± 14% for right trials. In comparison, the decrease in amplitude observed in the empirical results was 22 ± 12% in left trials and 27 ± 12% in the right trials. Thus, the simulated decrease in N1 amplitude was smaller than the empirically observed one, however this difference did not reach statistical significance [*F*_(1,17)_ = 3.7, *p* = 0.07]. This simulation suggested that a considerable part of the decrease in N1 amplitude observed in the average ERPs of the slower trials can be attributed to the higher N1 latency jitter observed in these trials. This is in line with the lack of correlation observed between single trial N1 amplitude and reaction time.

#### Independent Components’ Source Localization

**Figure 9:**
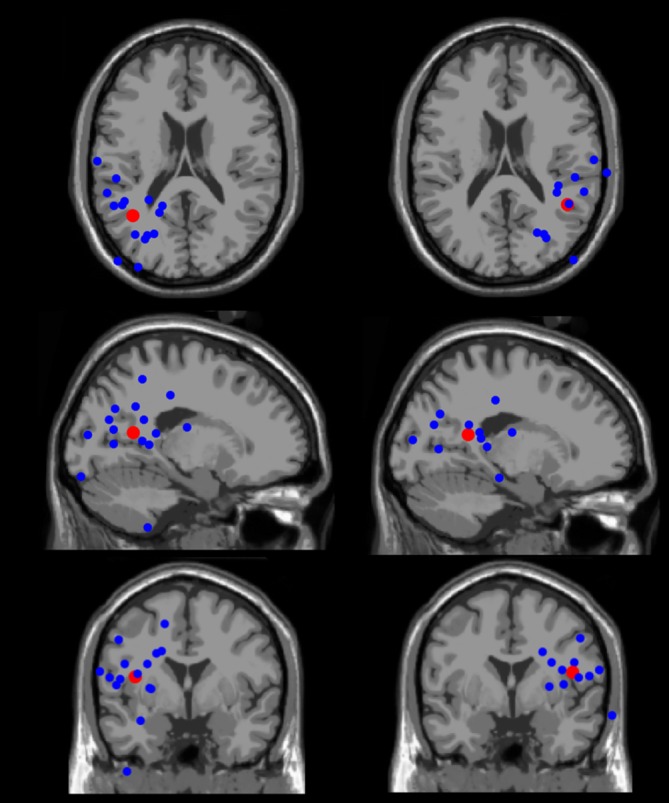
**Equivalent dipole locations.** The left and right hemisphere individual equivalent dipoles (blue) and the group-mean equivalent dipoles (red) are displayed in the left and right sides of the figure, respectively. The individual equivalent dipoles were localized laterally and posteriorly in the brain, in locations spanning the occipital, temporal, and parietal lobes. The group-mean equivalent dipoles were localized in the left and right superior temporal gyrus.

The bilateral mean of the equivalent dipoles source models was localized in the superior temporal gyrus of the left and right temporal lobes (Figure 9). Source localization of EEG data using equivalent current dipoles gives only an approximation of the actual locations of the cortical neural sources. We thus consider this result as an approximate location of the source of the neural processing studied here. The brain localization of the equivalent dipoles corresponding to the individual ICs were scattered across the occipital, temporal, and parietal lobes. Given the lateral and posterior location of the equivalent dipoles, these data suggest that the N1 potential analyzed originates in a high-level (extrastriate) visual area, probably belonging to the ventral stream.

## Discussion

Our single trial analysis of ERP data revealed how inter-trial variability in visual cortical responses predicts behavior from one moment to the next. Time point by time point analysis revealed several time windows after target onset that correlated significantly with reaction time. The earliest time point presenting a significant correlation with reaction time occurred at 175 ms just after the onset of the N1 visual evoked potential. Single trial N1 amplitude and latency analyses showed that inter-trial reaction time variability was related to single trial measurements of the latency of the N1 visual evoked potential but not to single trial N1 amplitude measurements. Equivalent dipole modeling indicated that the neural source of the N1 peak studied here was located in the temporal lobe. This location is compatible with high-level visual extrastriate areas. Earlier visual evoked responses (P1) were not found to be related with reaction time suggesting a top-down mechanism as the underlying cause of the variability in later visual processing.

Our single trial analyses of N1 peak amplitude and latency relied on the correct identification of this peak at the single trial level. This was likely facilitated by the effective separation by ICA of physiological responses from measurement noise, as suggested before De Vos et al. (2012). Visual inspection of the single trials component activity data suggested that the N1 peak could be identified in a large number of trials. Yet, in some trials this identification most likely failed. In order to reduce the impact of these outliers, we excluded the trials where the peak had been picked at the border of the search window, as has been done before Saville et al. (2015b), and given that these most likely do not represent local minima. In addition, we used robust statistics in our correlation analyses, thereby minimizing the effect of outliers (Pernet et al., 2013). Notably, the very significant correlation observed between single trial N1 peak latency and reaction time strongly suggests that peak identification was appropriate.

Our findings suggest that fast behavioral responses were associated with earlier activation of visual areas and slower behavioral responses with later activation. In contrast, although amplitude values varied substantially from trial to trial these held no predictive value with regard to reaction time. Notably, a recent study analyzing single trial neuronal responses in monkeys also observed a lack of correlation between the amplitude of extra-striate visual responses and behavioral latency (Lee et al., 2016). In fact, in their analysis of the neural code for behavioral latency, Lee et al. (2016) found that neural amplitude throughout the neural circuit, from sensory to motor areas, does not relate to behavioral response times. In line with our results, their study also revealed an important relationship between neural and behavioral latencies. This lack of correlation between the amplitude of neural responses and reaction time was surprising because previous studies suggested a relationship between peak amplitude and reaction time (Bahramali et al., 1998; Amano et al., 2006). However, these studies reported these differences in median-split averages. The higher latency jitter in the slower trials (associated with the higher reaction time variance in the slower half of the trials) might have contributed for the reduced amplitudes observed, just as in our tertile-split averages presented in Figures 3, 4, where the N1 peak of the slower trials appeared with reduced amplitude. Our simulation results confirmed this hypothesis showing that the higher N1 latency jitter observed in the slow reaction time tertile of trials results in a reduction in the N1 peak amplitude in the average ERP of similar scale as the one observed in the empirical results. This raises the hypothesis that the reported differences in amplitude levels of the N1 peak associated with different attentional states (e.g., Hopf et al., 2004) might also be, at least in part, due to increased latency jitter in the condition associated with slower reactions. An ERP component that has been the subject of latency jitter studies is the P3b ERP. P3b has been consistently found to be related to reaction time (Holm et al., 2006; Saville et al., 2011, 2012). P3b is a late event-related potential detected over centroparietal scalp sites in response to rare task relevant stimuli, and is considered to be a correlate of stimulus evaluation and categorization (Kok, 2001). P3b single trial studies demonstrated that trial-by-trial latency jitter leads to the under estimation of its peak amplitude in averaged responses (Holm et al., 2006; Saville et al., 2011), highlighting the importance of single trial measures when investigating inter-trial variability and correlations with behavioral performance. As for the N1 relationship described here, also, P3b single trial latency shows stronger correlation with reaction time than P3b amplitude (Holm et al., 2006; Saville et al., 2011), suggesting that the timing of these neural processes is more important to predict reaction time than their amplitude.

Functional MRI studies of neural correlates of reaction time intra-individual variability indicate a decrease in sensory cortex activation with increasing response times (Weissman et al., 2006, 2009). Notably, an increase in the latency of the sensory response might also be detected as a reduction in the BOLD signal at the early time points. Yet, given the low temporal resolution of the BOLD signal and the relatively small latency differences observed it is not obvious how these would be reflected at the level of BOLD activation.

Our observations suggested that the visual cortex fluctuates between periods of earlier stimulus processing (associated with fast reaction times) and periods where stimulus processing occurred at later time points. However, although very significant, the association between N1 peak latency and reaction time was relatively weak. N1 peak latency only accounted on average for around 10% of the reaction time variance. One possibility is that the increase in reaction time observed in the slow trials arises from a sum of the delay in stimulus processing with the delay in post-detection processing, including decision processes, motor preparation and execution, meaning that slow reaction times would be associated with a brain state where neural processing was slowed down at various stages including sensory processing. This possibility is compatible with the findings of Amano et al., 2006, that showed only a partial relationship between inter-trial reaction time variability and visual processing time estimated from MEG visual evoked responses, and is also in line with the fMRI findings that describe reaction time correlations in a distributed network of areas including parietal and frontal areas (Weissman et al., 2006, 2009). Furthermore, several EEG studies found a relationship between reaction time and late ERPs that occur close to the motor response over parietal and frontal areas (Kutas et al., 1977; Kida et al., 2003; Holm et al., 2006; Saville et al., 2011; Ramchurn et al., 2014; Bender et al., 2015). Interestingly, the relative contribution of each processing stage for reaction time variability may vary in different populations. In attention-deficit hyperactivity disorder (ADHD), Saville et al. (2015a) found a stronger relationship between the variability in motor related activity and reaction time variability, explaining the increased behavioral variability observed in this disorder.

Although our task did not require selective attention, i.e., did not require inhibition of distracters, the maintenance of attention towards the target visual features and location during task performance might have speeded up target identification and the motor responses. Slower responses might, thus, occur during periods where attention allocation moved away from the targets, slowing target processing. Weissman et al. (2006) findings suggest one such mechanism. In their study, participants performed a global/local selective-attention task where they were required to respond to one of two letters of hierarchically organized visual stimuli. Their findings suggest that modulation of bilateral inferior occipital cortex correlated with reaction time only for the congruent condition where the local letter and the global letter were the same and not for the incongruent condition. The authors interpreted this finding as an indication that reaction time fluctuates in synchrony with a general attentional mechanism that facilitates visual perception of both targets and distracters. Yet, in a multimodal study, Weissman et al. (2009) found that processing of targets and distracters were anti-correlated. In this second study, the authors investigated the relationship between reaction time and target/distracter processing in a task requiring identification of a visual target in the presence of an auditory distracter. Their findings suggested that slower responses were related to decreased activation of the visual cortex but increased activation of auditory cortex, thus further supporting the notion that slow trials are associated with periods where attention allocation moved away from the target. The trial-by-trial difference in sensory responses in these tasks are similar (but not necessarily the same) to the changes occurring due to attentional modulation in selective attention tasks. For example, in tasks requiring modulation of spatial attention, attending to one spatial location increases the sensory response and decreases reaction time to a stimulus located in the attended place (Corbetta and Shulman, 2002). Similar changes in sensory responses occur in response to goal-directed modulation of attention towards particular visual features or objects. In our study, we found that, using a task that did not require selective attention (in the absence of distracters), reaction time was also related to modulation of visual cortical responses. Although we did not monitor attention directly, in particular given that it can be separated into endogenous and exogenous components, it is possible that reaction time fluctuations might be a consequence of the attention focus moving from the visual sensory field to somewhere else (internal thoughts, somatosensory sensations, surrounding sounds). Indeed, as stated in the introduction, Weissman et al. (2006) showed that in comparison with fast trials, slow trials are associated with increased activation of the DMN, a network which activity correlates with periods of mind-wandering (Gruberger et al., 2011).

What are the mechanisms that underlie the fluctuations in the latency of sensory cortex responses? Previous studies investigating the neural correlates of reaction time variability found that reaction time could be predicted by pre-stimulus frontal brain activity patterns, both pre-stimulus activity levels (Weissman et al., 2006) and the pre-stimulus phase of brain oscillations (Drewes and VanRullen, 2011). The observed fluctuations in frontal lobe activity precede and might thus be the causal factor underlying the fluctuations in sensory responses, i.e., changes in frontal activity might affect the efficiency and the timing of sensory processing. A similar relationship has been described for attentional modulation of sensory responses where the frontal cortex has been shown to be necessary (Gregoriou et al., 2014). It is usually thought that high-level frontal areas control stimulus processing by enhancing sensory cortical responses, for example during attentional control (Corbetta and Shulman, 2002). As fluctuations in reaction time might originate from fluctuations in attentional control, our results suggest that fluctuations in attention are not necessarily related to the amplitude of the sensory responses but instead to their timing. It would be interesting to determine using single trial analysis, if this is also the case when comparing attended vs. non-attended conditions.

In conclusion, our study suggested that variability in the timing of extrastriate visual processing is related to reaction time variability. At the single trial level, N1 visual evoked potentials with early latencies were related to fast reaction times while later N1 latencies were associated to slow reaction times. In contrast, inter-trial variability in the amplitude of extrastriate visual responses was not related to reaction time variability, suggesting that, at least in our paradigm, behavioral response times are determined by the timing rather than the strength of the neural responses of the visual cortex. Although our data indicated a contribution of latency variability in visual processing to the variability in reaction time, it also indicated that this contribution is partial and that other mechanisms must also play a role. Our findings enhance our understanding of the neural correlates of intra-individual reaction time variability, and maybe of future importance for the characterization of the mechanisms that cause an increase in reaction time variability in individuals with frontal dysfunction, age-related cognitive decline, and disorders associated with attention deficits.

## Author Contributions

MJR, JSP and MC-B were involved in the conceptual phase and in the design of the study. MJR and JSP programmed the task, recruited the participants, acquired, and analyzed the data. MJR and MC-B worked on data interpretation. MJR drafted the manuscript, and MC-B and JSP revised it critically.

## Funding

This work was funded by Fundação para a Ciência e Tecnologia (Grants: SFRH/BPD/102188/2014, PD/BI/114377/2016, and UID/NEU/04539/2013-COMPETE, POCI-01-0145-FEDER-007440).

## Conflict of Interest Statement

The authors declare that the research was conducted in the absence of any commercial or financial relationships that could be construed as a potential conflict of interest.
