# Supplementary figures and images for: Spontaneous Fluctuations in Sensory Processing Predict Within-Subject Reaction Time Variability
Source: Front Hum Neurosci. 2016 May 9;10:200. doi: 10.3389/fnhum.2016.00200 (PMC4860412; doi:10.3389/fnhum.2016.00200)

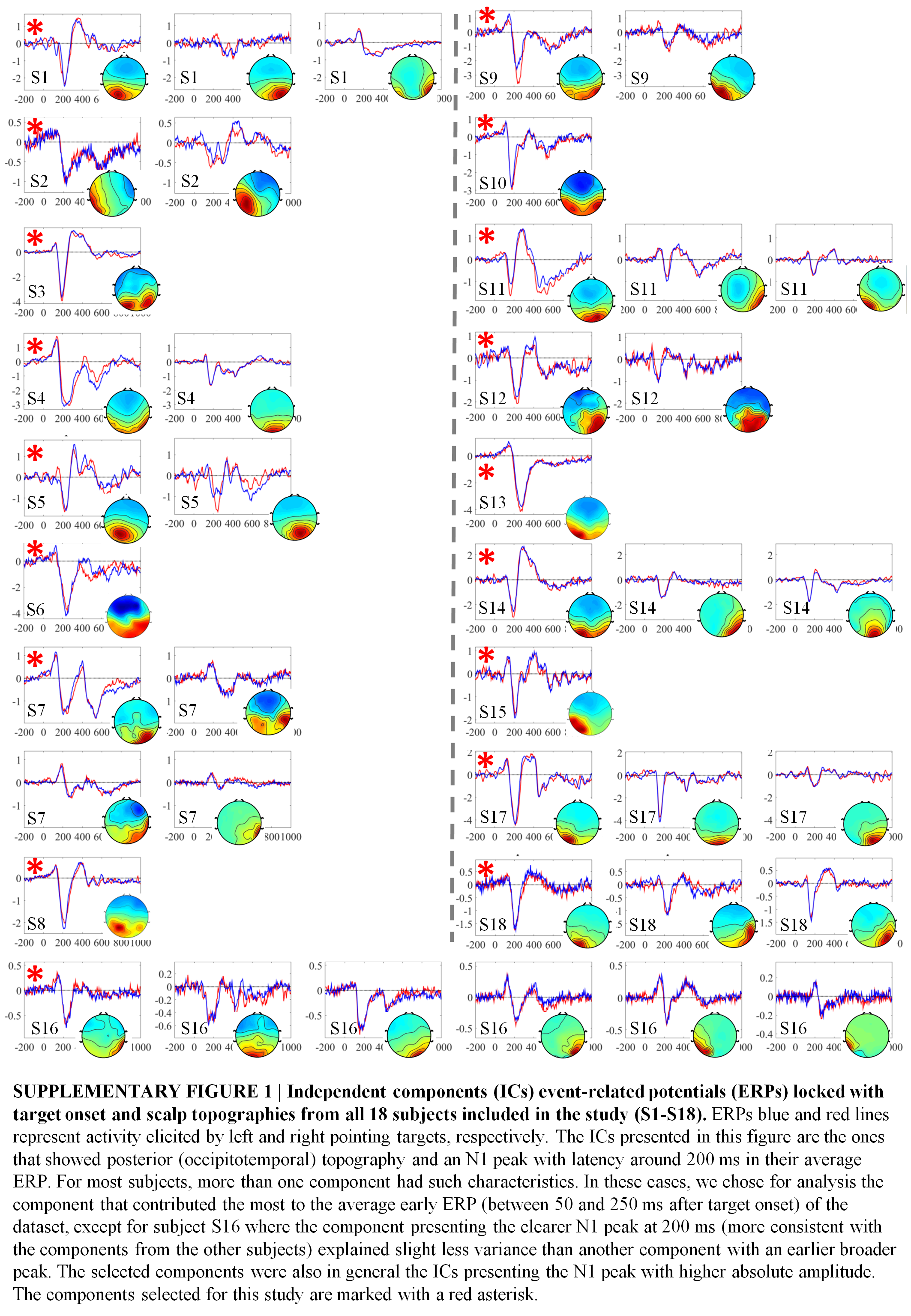

Supplement: Supplementary file 1 [file Image_1.tif]
